# Supplementary material for: Molecular and Antioxidant Characterization of Opuntia robusta Fruit Extract and Its Protective Effect against Diclofenac-Induced Acute Liver Injury in an In Vivo Rat Model
Source: Antioxidants (Basel). 2023 Jan 3;12(1):113. doi: 10.3390/antiox12010113 (PMC9855095; doi:10.3390/antiox12010113)
Supplement: Supplementary file 1 [file antioxidants-12-00113-s001.zip › antioxidants-2111986-supplementary.pdf]

Supplementary files

Table S1. Primer sequences for RT-PCR. All primers were designed using PrimerPlus3 (Cambridge, MA, USA) and BLASted in NCBI Nucleotide database (Bethesda, MD, USA)

Gene	Forward 5’→ 3’	Reverse 5’→3’
Actb	TGTCACCAACTGGGACGATA	GGGGTGTTGAAGGTCTCAAA
Sod1	CGGATGAAGAGAGGCATGTT	CACCTTTGCCCAAGTCATCT
Sod2	CCGAGGAGAAGTACCACGAG	GCTTGATAGCCTCCAGCAAC
Cat	CACCTGAAGGACCCTGACAT	CCATTCGCATTAACCAGCTT
Nrf2	GTCAGCTACTCCCAGGTTGC	ATCAGGGGTGGTGAAGACTG
Hmox-1	GAAGAAGATTGCGCAGAAGG	GAAGGCGGTCTTAGCCTCTT
Gclc	CACTGAGCTGGGAAGAGACC	GCCGCCATTCAGTAACAACT
Gadd45	TCTGTTGCGAGAACGACATC	TCCCGGCAAAAACAAATAAG
Nqo1	CCAATCAGCGCTTGACACTA	ACCACCTCCCATCCTTTCTT

Table S2. Compounds identified in *O. robusta* extract in positive ion mode

Compound	Formula	Adduct	*m/z*	Retention time (min)
**Lactones**				
D-glucaro-1,5-lactone	$C_6H_8O_7$	$M+NH_4$	210.0584	5.636
**Benzene and derivatives**				
Phenylethyl alcohol	$C_8H_{10}O$	M+H	123.0808	17.928
Methyl phenylacetate	$C_9H_{10}O_2$	M+H	151.0741	0.429
**Hydroxy acids and derivatives**				
(Â±)-Malic Acid	$C_4H_6O_5$	$M+NH_4$	152.0549	0.3005

Table S3. Compounds identified in *O. robusta* extract in negative ion mode

Compound	Formula	Adduct	*m/z*	Retention time (min)
**Organooxygen compounds**				
Glucose-1,3-mannose oligosaccharide	$C_{12}H_{22}O_{11}$	M+Cl	377.0885	0.919
Sucrose	$C_{12}H_{22}O_{11}$	M-H	341.1121	0.997
Fagopyritol B3	$C_{24}H_{42}O_{21}$	M-H	665.2215	0.997

Amylopectin	$C_{30}H_{52}O_{26}$	M-H	827.275	1.228
Maltohexaose	$C_{36}H_{62}O_{31}$	M-H	989.3259	1.306
Maltoheptaose	$C_{42}H_{72}O_{36}$	M-H	1151.377	1.357
beta-D-Xylopyranosyl-(1->5)-alpha-L-arabinofuranosyl-(1->5)-L-arabinose	$C_{15}H_{26}O_{13}$	3M-H	1241.398	1.357
Maltooctaose	$C_{48}H_{82}O_{41}$	M-H	1313.422	1.408
Maltononaose	$C_{54}H_{92}O_{46}$	M-H	1475.48	1.486
**Coumarins and derivatives**				
9-Hydroxy-4-methoxypsoralen 9-glucoside	$C_{18}H_{18}O_{10}$	M+FA-H	439.0885	0.919
**Imidazopyrimidines**				
Cis-zeatin	$C_{10}H_{13}N_{5}O$	M-H	218.1082	6.125
Gibberellin A94	$C_{19}H_{22}O_{6}$	M-H_2O-H	327.1286	14.605
